# Knowledge about and prevalence of *Chlamydia trachomatis* in a population-based sample of emerging Croatian adults

**DOI:** 10.1371/journal.pone.0293224

**Published:** 2023-10-27

**Authors:** Ivana Bozicevic, Tatjana Nemeth Blazic, Mirjana Lana Kosanovic Licina, Tatjana Marijan, Tomislav Mestrovic, Tihana De Zan, Aleksandar Stulhofer

**Affiliations:** 1 World Health Organization Collaborating Centre for HIV Strategic Information, University of Zagreb School of Medicine, Zagreb, Croatia; 2 Division for Epidemiology of Communicable Diseases, Department for HIV, Sexual and Blood Transmitted Diseases, Croatian Institute of Public Health, Zagreb, Croatia; 3 Division for Epidemiology, Department of Epidemiology of Infectious Diseases, Teaching Institute for Public Health “Andrija Stampar”, Zagreb, Croatia; 4 Division for Clinical Microbiology, Department of Molecular Microbiology, Teaching Institute for Public Health “Andrija Stampar”, Zagreb, Croatia; 5 University Centre Varazdin, University North, Varazdin, Croatia; 6 Department of Sociology Faculty of Humanities and Social Sciences, University of Zagreb, Zagreb, Croatia; University Medical Centre Ljubljana (UMCL) / Faculty of Medicine, University Ljubljana (FM,UL), SLOVENIA

## Abstract

To determine the prevalence of genital *Chlamydia trachomatis* (chlamydia) infection, knowledge about chlamydia and experience of previous testing for chlamydia, we carried out a national probability-based survey in emerging adults aged 18–25 years in Croatia in 2021–2022. Participants (*n* = 1197), members of a national online panel, completed a web-based questionnaire that collected information on socio-demographics, sexual behaviours and knowledge about sexually transmitted infections (STIs). Urine specimens from a sample of sexually experienced participants were self-collected and tested for chlamydia using Cobas 4800 CT/NG test. To achieve broad representativeness of the emerging adult population in the country, we applied post-hoc weighting for gender and age. Multivariable ordinary least squares linear regression was used to determine correlates of knowledge about chlamydia infection and binomial logistic regression to assess correlates of the willingness to test for chlamydia. Among 448 participants who sent in their urine specimens chlamydia prevalence was 2.5% (95% CI 1.2–5.1) in women and 1.0% (0.3–3.2%) in men. A total of 8.0% of women and 4.7% men reported testing for chlamydia prior to the survey. About a quarter of the sample was characterized by not answering correctly any of the six questions related to knowledge about chlamydia, while only 9.6% had five or six correct answers. In the multivariable analysis, significantly higher odds of willingness to test for chlamydia were found in females compared to males (*OR* = 1.34, *p* = 0.024), those with better knowledge about the infection (*OR* = 1.11, *p* = 0.005), and those with lower religiosity (*OR* = 0.91, *p* = 0.017). In conclusion, prevalence of chlamydia in emerging adults in Croatia is considerable. Efforts to control this infection should focus on primary prevention and targeted testing combined with effective case management strategies.

## Introduction

*Chlamydia trachomatis* (chlamydia) infection is one of the most frequent sexually transmitted infections (STIs) in young people and may cause complications such as pelvic inflammatory disease, ectopic pregnancy and infertility in women and epididymitis and epididymo-orchitis in men [1]. This infection is often asymptomatic, particularly in women, and can therefore remain undiagnosed and untreated. According to the World Health Organization (WHO) most recent estimates, prevalence of chlamydia among 15–49 years old population in the WHO European Region in 2016 was 2.7% (2.1–3.5%) [2].

In Croatia, the number of reported cases of chlamydia was declining in the 2011–2021 period, from the highest number of 386 cases in 2014 to 115 cases in 2021 while in this same time-period no new or enhanced chlamydia control activities were implemented at the national level [3]. According to the latest European Centre for Disease Prevention and Control (ECDC) data, chlamydia case notification rate in Croatia was 3.7 per 100,000 population in 2019 while the European Union and the European Economic Area (EU/EEA) average was 157.0 per 100,000 population [4]. Among 26 countries of the European Union and the EU/EEA that reported in the 2015–2019 period, the largest decline in the rate of reported cases was seen in Croatia (53%), compared to an average 6% decline in the EU/EEA countries. Differences in chlamydia reporting rates across Europe reflect availability of appropriate diagnostics, level of testing for chlamydia, data reporting practices of health care providers and intensity of chlamydia control activities [5, 6].

Cross-sectional surveys carried on in a representative sample of the general population can provide estimates of the burden of chlamydia provided that participation bias is not substantial, which is often not the case with household-based surveys of sexual behaviours and prevalence of STIs [7]. Recently, Internet-based surveys on sexual behaviours have been successfully utilised to recruit large samples of young people as Internet provides a private, anonymous setting for participation in research, which is beneficial for studies that involve reporting private and sensitive sexual information [8, 9].

The objective of our study was to determine the prevalence of chlamydia among 18–25 year old people in Croatia, assess the level of knowledge about chlamydia and experience of previous testing for chlamydia, and describe correlates of chlamydia-related knowledge and willingness to provide urine specimen to test for chamydial infection in the survey.

## Methods

### Participants

Data for this study were collected in a large-scale national sample of emerging Croatian adults. In the late 2021, 1,197 participants aged 18–25 years were surveyed using an online questionnaire. All participants were members of a commercial online panel maintained by an international research company. Such approach was deemed the most feasible and efficient considering the coronavirus disease 2019 (COVID-19) pandemic restrictions and widespread concerns that a household-based survey will yield a low response rate. Two-stage stratification (by region and settlement size) grid was used to randomly draw eligible participants from the panel database. Study response rate was 29%, which is comparable to the response of 32% observed in another national study of emerging adults’ sexual and reproductive health more than a decade ago [10]. To achieve broad representativeness of the emerging adult population in the country, we applied post-hoc weighting for gender and age.

### Procedure

Data collection, using computer-assisted web-survey, was carried out in from 19 November 2021 to 31 January 2022. Study design and procedures are in detail described elsewhere [11]. All participants were asked for informed consent before starting an online questionnaire and before participating in the biological part of the research. The questionnaire was originally developed in 2005 to assess knowledge about HIV and STIs, attitudes and beliefs about sexuality, and sexual behaviours of emerging adults [12]. The questionnaire was further validated in 2010, while a couple of recently developed measures were piloted in a sample of university students in 2020. The questionnaires took approximately 20 minutes to complete.

Following the completion of the questionnaire, for which they received a small token of appreciation (5 EUR voucher), participants were again contacted by the research company and offered to participate in a biological part of the study, which included provision of a urine sample for chlamydia testing. Participation in the biological arm of the study was rewarded with a voucher worth 20 EUR. Procedures in the process of sampling and communication with respondents (sending invitations to online questionnaire, testing kit, reminders to participate and view results) were carried out by the research company electronically using the usual way of communicating with panel members according to current regulations and existing standards in this area. The research was conducted in such a way that the anonymity of the participants towards the researchers and third parties was ensured. To preserve anonymity, randomly generated 4-digit codes were used to link biological and behavioral data. Testing kit was mailed via post along with a detailed photo-illustrated instructions and a link to a film about how to collect a urine specimen and check test results on-line. Participants were also provided with a specimen return package with the prepaid postage. Urine samples were mailed to the laboratory of the National Referent Center for the Diagnostics of STIs at the Zagreb Teaching Institute for Public Health “Dr. Andrija Stampar”. Specimens were analysed using Cobas 4800 CT/NG Test (Roche Diagnostics, Mannheim, Germany), which is a nucleic acid amplification test (NAAT) for detection of chlamydia.

Together with the testing kit, participants received a card with a unique code to be used for learning test result. Participant could obtain test results by typing in their personal code on a web page specifically designed for the purpose of the survey. Positive results were linked to information of health care specialists that were recommended to be contacted regarding start of the treatment.

All study procedures were approved by the Ethical Review Board of the Faculty of Humanities and Social Sciences, University of Zagreb (approval number 2019–14) and, for the biological arm, by the Institutional Review Board of the Croatian Institute of Public Health, (approval number 030-02/21-01/6 -381-15-20-3).

### Measures

The questionnaire included questions on demographics, sexual behaviours (number of sexual partners and condom use), knowledge about HIV and STIs and previous testing for chlamydia. *Knowledge about chlamydia infection* was assessed by six questions such as: “A person can get infected by chlamydia only once”; “Most women who are infected with chlamydia have no symptoms”, “In men, genital chlamydia infection can be without symptoms”, “If untreated, genital chlamydia infection can have negative consequences on fertility in men”, “If untreated, genital chlamydia infection can have negative consequences on fertility in women” and “Urogenital chlamydial infection can be detected from urine”. Answers were coded as 0 = incorrect and 1 = correct, and summed to form an additive indicator ranging from 0–6. The composite had acceptable internal consistency (KR-20 = 0.65).

Socio-demographics controls were participant’s education (years of formal education divided by age), participant’s family socioeconomic standing (ranging from 1 = “much worse than average family in the country to 5 = “much better than average family”), participant’s religiosity (i.e., frequency of attending religious ceremonies ranging from 1 = “I am not religious” to 7 = “daily or almost daily”, education of participant’s mother and father (0 = less than college education, 1 = college or university education), and place of residence (0 = urban, 1 = rural/semi-urban).

### Analytical strategy

Following descriptive analysis and t-testing for gender differences in key indicators, which were carried out on weighted data, two multivariable regression analysis were carried out: linear regression was used with knowledge about chlamydia infection as dependent variable and binomial logistic regression with willingness to test for chlamydia (non-tested vs. tested participants) as dependent variable. To check for potential underestimation of standard errors due to cluster-based sampling approaching, we first estimated intra-cluster correlation (ICC) in unconditional mixed models with sampling points as random effect. Considering that ICC for knowledge about chlamydia was 0.03 and ICC for testing for infection <0.01, the risk of false positives was judged highly unlikely. The proportion of missing data on indicators of interest was low (up to one percent), with the notable exception of the variable number of sexual partners in the past 12 months, which had 31% of missing responses. Following Little’s test of missing completely at random, which suggested that data were missing in non-systematic manner (χ^2^_(20)_ = 27.19, *p* = 0.130), we applied multiple imputation (*m* = 40) to check the robustness of regression findings [13].

To avoid potential biases associated with an overly liberal approach to the inclusion of control variable, only constructs that have been both conceptually and empirically linked to the outcomes (family socioeconomic background, parents’ education, religiosity, and rural vs. urban place of residence) were included in the regression models, together with the number of sexual partners in the past 12 months as an indicator of sexual risk taking, knowledge about chlamydia, as well as previous testing for the infection [14, 15]. Finally, gender was controlled due to gender-specific sexual socialization of young people and higher levels of health concerns in women, especially in the context of reproductive health, compared to men.

Statistical analysis was carried out using IBM SPSS Statistics for Windows, version 29 (IBM Corp., Armonk, N.Y., USA) (descriptive, bivariate, and multivariable analyses with post-hoc weighting) and jamovi statistical software packages (ICC testing) [16].

## Results

Socio-demographic characteristics of the sample are presented in Table 1. Mean age of participants was 21.7 years (*SD* = 2.21) and 48.3% of participants were female. Reflecting national educational structure, most participants reported that their parents completed secondary education. The majority reported some level of religiosity. Regarding sexual behavior, most participants (74.6%) reported a single sexual partner in the past 12 months. Eight percent had no partners in the same period, while 10.8% reported two and 6.6% three or more sexual partners.

**Table 1:** Basic sociodemographic information about respondents (unweighted n = 1197 and weighted n = 1198 data).

	Unweighted	Weighted
	*n* (%)	*n* (%)
Age		
18–21	423 (35.3)	554 (46.2)
22–25	774 (64.7)	644 (53.8)
Gender		
female	606 (50.6)	597 (48.3)
male	591 (49.4)	619 (51.7)
Mother’s education		
primary	110 (9.2)	113 (9.4)
secondary	775 (65.0)	763 (64.1)
tertiary	307 (25.8)	316 (26.5)
Father’s education		
primary	119 (10.0)	122 (10.2)
secondary	777 (65.5)	776 (65.3)
tertiary	291 (24.5)	291 (24.5)
Family socioeconomic standing		
Much worse than the average family	22 (1.8)	24 (2.0)
Somewhat worse than the average family	111 (9.3)	109 (9.1)
Comparable to the average family	751 (62.7)	762 (63.6)
Better than the average family	281 (23.5)	274 (22.9)
Much better than the average family	32 (2.7)	29 (2.4)
Religiosity (religious ceremonies attendance)		
not religious	155 (12.9)	145 (12.1)
never	124 (10.4)	123 (10.3)
once a year or less often	193 (16.1)	194 (16.2)
several times a year to once a month	538 (44.9)	540 (45.2)
weekly to daily	187 (15.7)	196 (16.3)
Place of residence		
rural	451 (37.7)	461 (38.5)
urban	746 (62.3)	737 (61.5)
Ever tested for *Chlamydia trachomatis*		
no	1012 (84.5)	1030 (86.0)
yes	93 (7.8)	75 (6.3)
do not remember	92 (7.7)	92 (7.7)

A total of 553 participants (46.3% of the sample) expressed interest to receive urine specimen collection device. The majority (81.0%) returned a urine sample. Of the 448 emerging adults who sent in their urine sample—40.4% (n = 245) of female and 34.3% (n = 203) of male participants -, positive result was obtained in nine participants–seven women (2.5%; 95% CI 1.2–5.1) and two men (1.0%; 0.3–3.2). All nine individuals who tested positive learned about their result. According to the weighted analysis, only 6.3% of participants (8.0% of female and 4.7% of male participants) tested for chlamydia prior to this survey while an additional 7.6% of females and 7.8% of males did not remember.

Knowledge about the infection, which ranged from 0 (no correct answers) to 6 (all sex questions correctly answered), was skewed toward lower knowledge (*M* = 2.2 *SD* = 1.65; Median score = 2). About a quarter of the sample was characterized by not a single correct answer, while only 9.6% had five or six correct answers. Most emerging adults either reported no (21.6%) or only three correct answers (21.6%). We observed no substantial gender difference in knowledge about the infection (*t*_(1196_ = -1.72, *p* = 0.086). While approximately one in two respondents knew that infection with chlamydia can have adverse consequences on female and male fertility, only 4.5% of men and 1.9% of women knew that this infection can be acquired more than once in a lifetime. A minority– 24.6% of male and 25.2% of female respondents—knew that the majority of women with chlamydia infection do not have any symptoms.

### Correlates of knowledge about and testing for chlamydia

Tables 2 and 3 show correlates of knowledge about chlamydia and of providing urine specimens for testing, respectively, using weighted multivariable regression analysis. The results of multivariable regression analysis suggest that participants who self-reported being tested for chlamydia before this survey had significantly better knowledge about chlamydia (*B* = 0.87, S.E. = 0.21; p<0.001) compared to those who did not test before.

**Table 2:** Predictors and correlates of knowledge about chlamydia (*n* = 921).

	*B*	S.E.	*p*
Gender (male = reference)	0.15	0.11	0.182
Age-adjusted years of education	0.85	0.67	0.205
Family socioeconomic standing	0.02	0.08	0.815
Urban-rural dwelling (rural = reference)	-0.01	0.12	0.923
Mother’s education (less than college = reference)	-0.23	0.14	0.088
Father’s education (less than college = reference)	-0.08	0.14	0.591
Religiosity	-0.06	0.04	0.096
Number of sexual partners in the past 12 months	-0.10	0.08	0.251
Ever tested for *Chlamydia trachomatis* (no = reference)	0.87	0.21	0.000

S.E. = Standard error

**Table 3:** Predictors and correlates of chlamydia testing (*n* = 921).

	OR	95% CI
Gender (male = reference)	1.24	0.93–1.64
Age-adjusted years of education	1.84	0.35–9.81
Family socioeconomic standing	0.98	0.80–1.22
Urban-rural dwelling (rural = reference)	1.05	0.78–1.40
Mother’s education (less than college = reference)	1.35	0.96–1.90
Father’s education (less than college = reference)	0.85	0.59–1.21
Religiosity	0.94	0.85–1.03
Number of sexual partners in the past 12 months	1.13	0.92–1.39
Ever tested for chlamydia (no = reference)	1.40	0.84–2.32
Knowledge about chlamydia	1.08	0.99–1.17
	OR^a^	95% CI^a^
Gender (male = reference)	1.32	1.03–1.70*
Age-adjusted years of education	1.45	0.35–6.08
Family socioeconomic standing	1.05	0.88–1.25
Urban-rural dwelling (rural = reference)	0.90	0.70–1.15
Mother’s education (less than college = reference)	1.28	0.95–1.73
Father’s education (less than college = reference)	0.87	0.64–1.19
Religiosity	0.91	0.84–0.98*
Number of sexual partners in the past 12 months	1.09	0.90–1.33
Ever tested for chlamydia (no = reference)	1.30	0.84–2.02
Knowledge about chlamydia	1.11	1.03–1.19**

* *p* < 0.05,

** *p* < 0.01;

^a^ Estimates obtained on dataset in which missing data were replaced by multiple imputation (*m* = 40)

OR = Odds ratio; CI = confidence interval

Table 3 (the upper part) shows the results of testing for correlates of providing a urine sample. Somewhat unexpectedly, none of the independent variables significantly contributed to distinguishing between emerging adults who tested for chlamydia in the current study and those who did not.

In the final step, the two regression analyses were repeated using the dataset in which missing information was replaced by multiple imputation. In the case of knowledge about chlamydia (not shown in a table), ever being testing for the infection remained the sole significant predictor (*B* = 0.74, S.E. = 0.18, *p* < 0.001). However, the second analysis resulted in a markedly different pattern of (non-)significant findings (see the lower half of Table 3). With missing data imputed, three significant predictors of testing for chlamydia emerged: gender (*OR* = 1.34, *p* = 0.024), knowledge about the infection (*OR* = 1.11, *p* = 0.005), and religiosity (*OR* = 0.91, *p* = 0.017). While female gender and better knowledge about the infection significantly increased the odds of testing, higher religiosity worked in the opposite direction.

## Discussion

The prevalence of chlamydia infection in our study was relatively similar to the prevalence found in nationally representative surveys of sexually experienced adults aged 18–26 years in the EU/EEA countries (3.6%) in women, but lower in men (3.5% in the EU/EEA) [5]. The prevalence found in this survey was substantially lower compared to the last round of nationally representative survey among adults aged 18–25 years in Croatia, which found prevalence of chlamydia of 5.3% in women and 7.3% in men in 2010 [17]. However, that study was household-based, with a lower response rate for testing for chlamydia (32.5%) compared to this round of the survey (46.2%). Comparisons between these two surveys should be made with caution due to differences in the recruitment strategies (previous survey was household-based while this one was internet-based) and the fact that the surveys were not powered to detect a change in prevalence. There is evidence that estimates of chlamydia prevalence might be higher in surveys with lower response rates compared to surveys with higher response rates [5].

Prevalence of chlamydia in our study was similar to the prevalence of 2.99% among 1238 students in the city of Zagreb who were tested for chlamydia as part of a three-year screening conducted in in 2017–2019 [18].

Only a minority of respondents in our survey reported being tested for chlamydia before, which shows low availability of chlamydia testing in Croatia. A substantially higher proportion of respondents aged 16–24 years sampled in the nationally representative survey in Britain (Natsal-3) reported being tested for chlamydia in the year before the survey– 54.2% of women and 34.6% of men, reflecting longer-standing and extensive presence of screening for chlamydia in Britain [19]. Furthermore, in a study on randomly selected 377 women aged 18–25 from Washington State in the US, 53% self-reported chlamydia testing in the preceding year [20].

It is discouraging to observe that those with higher number of partners in the past 12 months were not significantly more likely to provide urine specimens for testing for chlamydia in the survey, which might indicate low perception of risk of infection or the possibility that the survey sampling method did not reach those at higher risk of infection.

In contrast to that, those with better knowledge about chlamydia were significantly more likely to provide urine samples for testing. This shows that interventions focused on increasing knowledge and awareness about chlamydia could lead to better uptake of testing.

There is no organized opportunistic testing for chlamydia in Croatia. ECDC recommends that widespread opportunistic testing or screening of sexually active men and women under 25 years in case of sufficient resources and existence of a monitoring and evaluation system, none of which is in place in Croatia [21]. However, effectiveness of chlamydia screening on lowering chlamydia prevalence, preventing pelvic inflammatory disease, ectopic pregnancy or female infertility has been questioned and is a subject of ongoing debates [22, 23]. Targeted testing combined with effective case management strategies, which includes partner notification, and the emphasis on primary prevention of STIs might be a reasonable way forward for chlamydia control in Croatia [20, 24, 25]. It is also important to consider participation rates in such testing approaches, since de Wit and colleagues showed that they are crucial for reaching (or preserving) cost-effectiveness of nationwide chlamydia screening programs–emphasizing the need for extensive piloting prior to implementation decisions [26].

We found considerable gaps in knowledge about chlamydia among young people. Therefore, there is a need for raising awareness and knowledge in the general population of young people about asymptomatic nature of chlamydia infection and its consequences and the risks of re-infections, which might lead to higher demand for testing [27].

Establishment of online services for chlamydia testing should be also considered as this may address barriers to clinic attendance such as long waiting times, inconvenient opening hours, perceived stigma and travel time or cost. Availability of online services in the community alongside clinic services can increase uptake of STI testing in young people, and in particular those that have lower access to services [28], while the experience from Sweden emphasized behavioural risk profile and antecedent chlamydia test results in users of such services to demonstrate how it can indeed reach a relevant target group [29].

### Study limitations

Our findings are impacted by accuracy of self-reporting on sexual behaviours. Although Internet recruitment into surveys has a number of advantages it is affected by the sampling and participation bias, which is especially relevant in research on sexual behaviours due to their private nature. Questions were answered via internet and by self-completion, which should have minimised social desirability bias. Since data collection was done on-line, the survey included population with access to internet via mobile or desktop devices. According to data of the Croatian Bureau of Statistics, 86% of households had access to Internet in 2021, while 97% of people aged 16–24 years used Internet [30].

Regarding chlamydia testing, first catch urine is an adequate specimen for screening high-risk groups such as emerging adults for chlamydia by NAAT [31]. COBAS 4800 test used in our study is validated for first catch urine testing in men and women, while transport media used in the testing procedure ensures cellular quality and improves the sensitivity.

Besides its limitations, the internet-based survey on sexual behaviours, which included self-collection of urine samples and testing for chlamydia in young people in Croatia has proven to be feasible and a lower-cost alternative to a household-based survey. It has also been a powerful tool to update and fill the gap of information concerning the prevalence of chlamydia among emerging adults and knowledge about chlamydia infection.

In conclusion, our study found a considerable prevalence of chlamydia among young people in Croatia and low reported prior testing, which implies a need to strengthen chlamydia control efforts by providing innovative targeted chlamydia screening strategies.

## Supporting information

S1 Data (SAV)
